# Is the risk of low birth weight or preterm labor greater when maternal stress is experienced during pregnancy? A systematic review and meta-analysis of cohort studies

**DOI:** 10.1371/journal.pone.0200594

**Published:** 2018-07-26

**Authors:** Silvana Andréa Molina Lima, Regina Paolucci El Dib, Meline Rossetto Kron Rodrigues, Guilherme Augusto Rago Ferraz, Ana Claudia Molina, Carlos Alberto Pilan Neto, Marcelo Aparecido Ferraz de Lima, Marilza Vieira Cunha Rudge

**Affiliations:** 1 Nursing Department, Botucatu Medical School, UNESP–Univ Estadual Paulista, Botucatu, Brazil; 2 Institute of Science and Technology, Department of Biosciences and Oral Diagnosis, UNESP, São José dos Campos, Brazil; 3 Nursing Department of Gynecology and Obstetrics, Universidade Estadual Paulista (Unesp) Botucatu Medical School, UNESP, Botucatu, Brazil; 4 Municipal Authority of Botucatu, São Paulo, Brazil; 5 Minas Gerais Medical School, UFMG -Univ Federal de Minas Gerais, Belo Horizonte, Brazil; 6 Department of Collective Health, Botucatu Medical School, Botucatu, São Paulo, Brazil; University of Washington, UNITED STATES

## Abstract

Antenatal stress is linked to fetal risks that increase the chances of neonatal complications and reduction of child cognitive ability. Therefore, we aimed to evaluate if maternal stress affects fetal, neonatal or child development. The following databases were searched: MEDLINE (1966 to May 2016), Embase (1980 to May 2016), LILACS (1982 to May 2016) and CENTRAL (1972 to May 2016). Observational studies published in English and Portuguese were included whether there was any relationship between fetal and neonatal outcome, such as birth weight, preterm labor, child development with pregnant women that were subjected to any stress type during at least one month of follow-up. Two independent reviewers screened eligible articles, extracted data and assessed the risk of bias. Thus, 8 cohort studies with about 8,271 pregnant women and 1,081,151 children proved eligible. Results suggested a significant association between antenatal stress exposure and increasing rates of low birth weight (Odds ratio (OR) 1.68 [95% Confidential Interval (CI) 1.19, 2.38]). However, there was no statistically significance difference between non-exposed and exposed groups related to preterm labor (OR 1.98 [95% CI 0.91 to 4.31]; I2 = 68%, p = 0.04). Although, results were inconsistent with primary analysis suggesting a significant association between antenatal stress exposure and the occurrence of higher rates of preterm birth (OR 1.42 [95% CI 1.05 to 1.91]; I2 = 68%, p = 0.04) in the sensitivity analysis. Furthermore, the current review has suggested that stress perceived during antenatal negatively influences fetal life and child development. Yet, further studies are necessary with adequate sample size and longer follow-up time to confirm our findings.

## Introduction

It is known that stress is cause for many diseases under urban environment, since it is a physiological response to the mental, emotional or physical challenges that we experience [1].

The stress causes the immediate and long-term disturbance in the psychoneuroendocrine and immunological pathways. The hypothalamus pituitary adrenal (HPA) axis and sympathetic nervous system gets adversely affected and hyperactivated by influx of emotions from limbic system under mental stress; therefore, increasing the release of cortisol and catecholamine hormones [2]. Furthermore, the repeated initiation of the ‘fight or flight’ response can lead to a dysregulation of sympathetic nervous system and HPA axis, consequently compromising the homeostasis [2,3].

In stress event, a passive response releases high levels of corticotropin hormone (CRH) into hypothalamic paraventricular nucleus. CRH acts on pituitary, stimulating the release of adrenocorticotropin hormone (ACTH). ACTH acts on adrenal glands and increases glucocorticoids production (i.e. cortisol in humans and primates; corticosterone in rodents). An increase in glucocorticoid levels is responsible for several metabolic and physiological changes that are important in stereotyped stress. Furthermore, hypothalamic-pituitary-adrenal axis is closely related to immune system through lymphocyte cells, that is, they produce immunosuppression that is a response of stress [2,3].

Bearing in mind that hypothalamus also connects to this axis, as a result there are several behavioral changes under stress situations [2,3], such body response is related to a ‘fight mode’ to deflect threatening situations, since SA axis is activated in response to stress, whenever there is a challenge. The disturbances caused by activation of the HPA axis during pregnancy are identified as those responsible for changes found during mothers offspring subjected to some stress type [4]. Therefore, HPA axis activation alters regulatory neurotransmitters levels and distribution, that is, norepinephrine, dopamine, serotonin and acetylcholine. Besides that limbic system structure changes mother´s behavior and morphology [5,6,7].

Current literature relates stress during pregnancy, but there is paucity in literature on effects of antenatal stress, since cognitive development is challenging to be assessed, as participants are usually facing many stress types [8].

During pregnancy, moderate to severe life stress and maternal anxiety [5] increase the risk of fetal distress, prematurity, low birth weight, neonatal crying [9], acute health problems in the first year of life [10], as well as behavioral and emotional problems until the age of four [11].

Antenatal stress is linked to cognitive and neuromotor development reduction, in addition to child's behavior inhibition [1,2]. Although, biological responses related to environmental and psychological stress is a disorder that has been already known, it is not clear the consequences in humans. Therefore, we aimed to evaluate if maternal stress exposure affects fetal, neonatal (birth weight or preterm labor) or child development throughout a systematic review of cohort studies.

## Methods

Our reporting adheres to the Meta-analysis of Observational Studies in Epidemiology (MOOSE) Statements [12].

### Eligibility criteria

The current review included cohort studies with a follow-up of at least one month. Studies were only included if pregnant women were subjected with any stress type, regardless of their age.

During antenatal care, maternal stress could be related to environment (i.e. nature disaster and work related); physiological (i.e. chest pain, irritability, cardiac palpitations); emotional (i.e. memory loss, nightmares, inability to concentrate, accident, relative loss). There are several tools to measure stress according to the included studies, such as validated self-report questionnaires (i.e. Nursing Stress Scale [13], anxiety and depression, the State Trait Anxiety Inventory [14], General Health Questionnaire [15], Beck Depression Inventory [16]); perceived stress self questionnaire; physical symptoms and physiological parameters (i.e. hormone levels, such as prolactin, corticosteroids or others). Control groups were defined with no stress exposure or low stress level by the included studies.

Potential confounders were related to previously mother health problems, such as diabetes; cardiovascular diseases; smoking during pregnancy; mental health problems (i.e. major depression); using contraindicated medication during pregnancy; maternal age (<27, 27–30, 31 years and over); socioeconomic status (maternal income and education) and twin pregnancy.

The primary outcomes of interest were fetal (measured in utero and/or at birth) and infant growth; birth outcomes such as low birth weight, still birth or preterm birth or low APGAR scores that required resuscitation at birth; physical abnormalities (i.e. congenital malformation); developmental and behavioral outcomes, such as cognitive and learning functions; and childhood overweight.

### Data source and searches

Pertinent literature was identified through PubMed (from 1966 to March 2016); Embase (from 1980 to March 2016); LILACS (from 1982 to March 2016); and CENTRAL (up to March 2016), regarding to studies that associated fetal, neonatal and children development with maternal stress perceived during pregnancy with at least one month of follow-up. The data gathering was restricted to Portuguese and English-language studies. There were no publication status restrictions. Moreover, the last search took place on May 05, 2016; and search strategy is presented in S1 Appendix. Besides reference list of relevant studies is scrutinized for further citations. PRISMA Checklist is presented in S1 Table.

### Selection of studies

**Fig 1:**
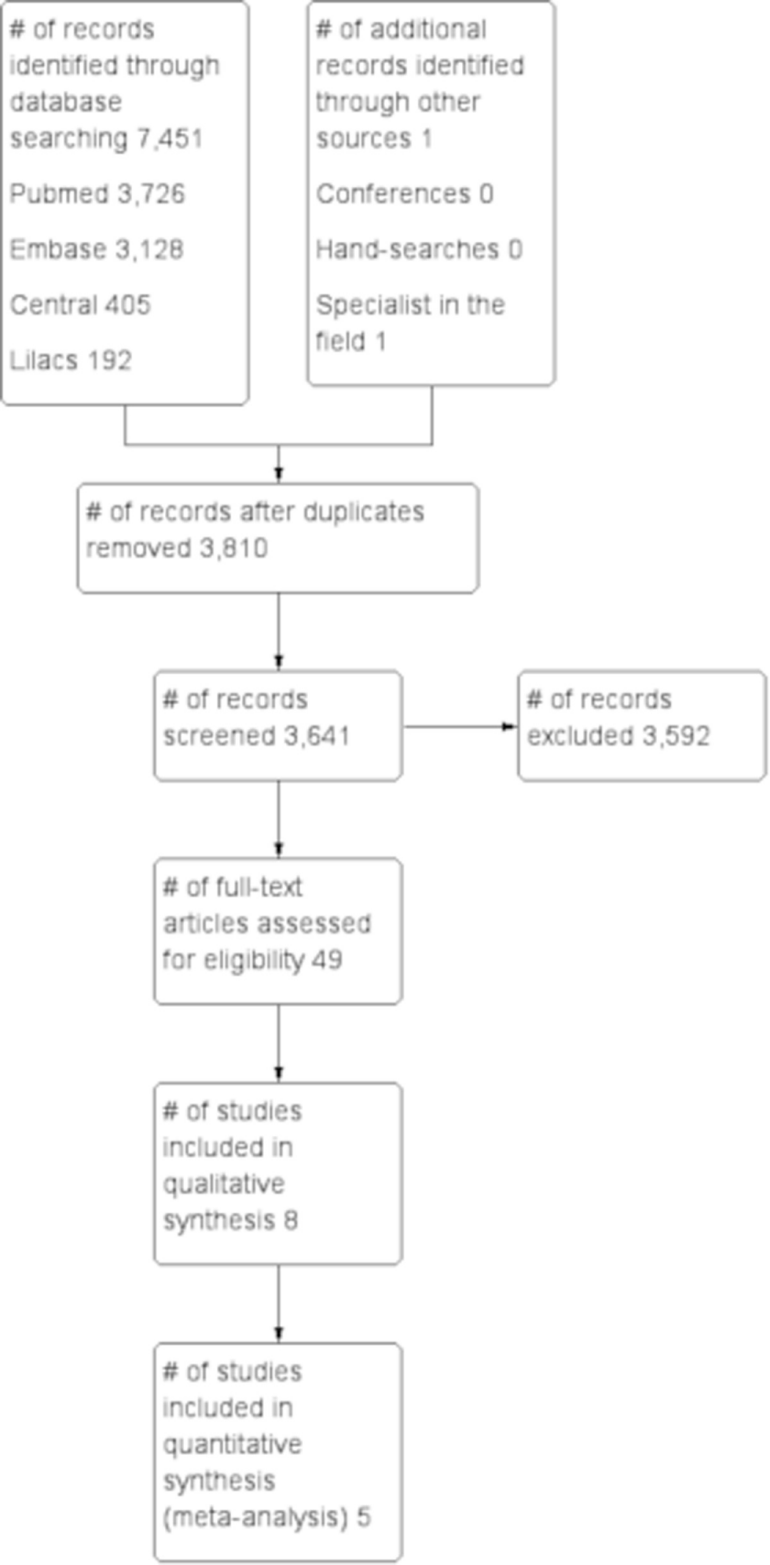
Flowchart of the review.

Two independent reviewers (MR and GARF) screened all titles and abstracts that were identified through literature search. Moreover, they selected potential studies by obtaining the full-text articles, and then evaluated them, in accordance with the eligibility criteria. The study selection flowchart was expressed in Fig 1.

### Data extraction and risk of bias assessment

Two independent reviewers (MR and GARF) screened all the potential quantitative results or critical data from some preselected studies, with regard to the participants, stress type, control conditions, outcome measurements and results. Subsequently, disagreements between the reviewers were discussed with other two authors (SM and RED) to reach consensus.

For cohort studies, reviewers independently assessed risk of bias with a modified version of the Ottawa-Newcastle instrument [17] that includes confidence in assessment of exposure and outcome, adjusted analysis for differences between groups in prognostic characteristics, and missing data [18]. For incomplete outcome data, we stipulated that low risk of bias consisted of loss to follow-up of less than 10% and a difference in missing data between exposure and control groups of less than 5%.

### Data synthesis and statistical analysis

We calculated 95% confidential intervals (CI) around odds ratios by using RevMan software to combine results in a forest plot of random effect model. Although, we only used fixed effect model if there was non-statically significance difference, considering sensitivity analysis. We planned to perform subgroup analysis for stress time during pregnancy; degree of perceived stress and stress type (physical versus psychological), but there were not enough studies.

Authors of included studies were contacted whether there was a need for further attempts to request or analyze unpublished data. If there was no response or there was response but could not provide data, such outcomes were excluded from analysis. Furthermore, studies with missing outcomes were described within studies characteristics table.

### Investigation of heterogeneity

Heterogeneity of the studies was explored within Chi^2^ test and I^2^ value [19] that provides relative amount of variance of summary effect due to between-study heterogeneity. We classified heterogeneity using the following I^2^ values: 0 to 40%: might not be important; 30% to 60%: may represent moderate heterogeneity; 50% to 90%: may represent substantial heterogeneity; and 75% to 100%: considerable heterogeneity.

## Results

### Study selection

A total of 7,451 titles were identified in the databases cited above, but only 49 studies were selected for detailed evaluation. After assessing the full articles, 37 publications were considered for inclusion, but 30 studies [20–49] were excluded, since they were case-control, cross-sectional or prospective studies without control group. Ultimately, it was found that only eight studies [50–56] that included 8,271 pregnant women and 1,085,151 children were eligible for the current review (Table 1).

**Table 1:** Search strategy.

(Maternal OR (Maternal Exposures) OR (Maternal Exposure) OR (Maternal Ages) OR (Maternal Age) OR Mother OR Mothers OR(Mothers' Clubs) OR (Mothers' Club) OR (Mother Clubs)) **AND** ((prenatal maternal stress) OR (Physiological Stresses) OR (Physiological Stress) OR (Metabolic Stress) OR (Metabolic Stresses) OR Stress OR (Physiological Stress Response) OR (Physiological Stress Responses) OR (Physiological Stress Reactivity) OR (Physiological Stress Reaction) OR (Physiological Stress Reactions) OR (Biological Stress) OR (Biological Stresses) OR (Metabolic Stress Response) OR (Metabolic Stress Responses) OR (Stress Response) OR (Stress Responses) OR (Psychological Stresses) OR (Life Stress) OR (Life Stresses) OR (Psychologic Stress) OR (Psychological Stress) OR (Mental Suffering) OR Suffering OR Anguish OR (Emotional Stress) OR (Post-Traumatic Stress Disorder) OR (Post Traumatic Stress Disorders) OR PTSD OR (Posttraumatic Neuroses) OR (Posttraumatic Stress Disorders) OR (Posttraumatic Stress Disorder) OR (Post-Traumatic Neuroses) OR (Post Traumatic Neuroses) OR (Chronic Post-Traumatic Stress Disorder) OR (Chronic Post Traumatic Stress Disorder) OR (Delayed Onset Post-Traumatic Stress Disorder) OR (Delayed Onset Post Traumatic Stress Disorder) OR (Acute Post-Traumatic Stress Disorder) OR (Acute Post Traumatic Stress Disorder) OR (Heat Stress Disorder) OR (Heat Stress Disorders) OR (Heat Stress Syndromes) OR (Heat Stress Syndrome) OR (Heat Cramps) OR (Heat Cramp) OR (Acute Stress Disorder) OR (Acute Stress Disorders) OR (Traumatic Stress Disorder) OR (Traumatic Stress Disorders) OR (Cold Shock Response) OR (Cold-Shock Responses) OR (Cold-Stress Reaction) OR (Cold Stress Reaction) OR (Cold-Stress Reactions) OR (Cold-Stress Response) OR (Cold Stress Response) OR (Cold-Stress Responses) OR (occupational stress))

Three of these studies presented a prospective inception cohort design [51,52]; a mix of cohort and case-control studies were authored by Li 2010a [53] and Li 2010b [54]. Chuang 2011[50]; Whitehouse 2010 [55]; Xiong 2008 [56] and Tandu-Umba 2014 [57] studies were classified as prospective cohort designs. Sample size varied from 186 to 3,531 women; and 299 to 1,015,910 children (Table 2).

**Table 2:** Study characteristics related to number of participants, inclusion and exclusion criteria.

Author, year	No.*participants	Inclusion criteria	Exclusion criteria
Umba 2014 [57]	Women: 1,082 Children: NR	Women having given birth during a 6-month period (from 15 February 2013 throughout 15 August 2013) at 13 biggest maternities of Kinshasa, the capital of DR Congo	Multiple pregnancies; non-consenting women; and those failing to answer some items.
Everard 2011 [52]	Women: 3,531 Children: NR	Mulliparous; low risk women, who participated in the SCOPE (Screening for Pregnancy Endpoints) study	Not reported
Chuang 2010 [50]	Women: 186 Children: NR	Pregnant women going to for delivery and postpartum care during April 2004 to January 2005. Only term born infants (gestational weeks 37 and birth weight 2500 g).	Not reported
Li 2010a [53]	Women: NR Children: 1.015,910	All singletons born between January 1987 and 31 December 2001.	Children with autism and with mental retardation
Li 2010b [54]	Women: NR Children: 65,671	All live born children and new residents in Denmark.	Not reported
Whitehouse 2010 [55]	Women: 1,309 Children: 1,309	Gestational age between 16 and 18 weeks, English language skills sufficient to understand the implications of participation, an expectation to deliver at KEMH, and an intention to remain in Western Australia to facilitate future follow-ups of their children.	Children whose language development may be compromised due to speaking a language other than English at home; children who had Downs syndrome, autism or intellectual disability with a known cause; and aboriginal mothers.
Xiong 2008 [56]	Women: 301 Children: 299	English speaker; planning to deliver at the study hospitals; being over 18 years old; (for New Orleans) living in the area before the storm; and (for Baton Rouge) not having an extensive experience of the hurricane (evacuating or having a relative die).	Not reported
Dole 2003 [51]	Women: 1,943 Children: 1,962	Women from two prenatal clinics in central North Carolina, who were between 24 and 29 weeks’ gestation, beginning in August 1995. Only preterm children were assessed (<37 weeks)	Could not speak English; age<16 years; a multiple gestation; did not plan to deliver at the study site; or lacked telephone access.

*No.: number at baseline.

Additionally, maternal age ranged from 13[53] to 35 years [56] (Table 3); children were followed up from 0 to 13 years in Australia [55], USA [51, 56], Denmark [53, 54], Taiwan [50], England [52] and Africa [57] (Table 3). Only one study recruited participants from Screening for Pregnancy Endpoints (SCOPE), but there was no record of the place [52] (Table 3).

**Table 3:** Study characteristics related to setting; number of participants according to the group; maternal age; definition of stress, outcome measures for the women and the children; and follow up.

Author, year	Location	No.*participants per group	Maternal age (mean)	Definition of Stress	Outcome measures	Follow-up
Umba 2014 [57]	Congo	Stressed women: 618 Non-stressed women: 464 Children:NR	28	Stress factors that built differences between stressed and non-stressed women were unplanned pregnancy, much desired pregnancy; preciousness; previous prematurity; relative’s illness/death; partner´s illness/death; and tension in couple/family.	Maternal: Stress scores were established using 2 tools: 1) perceived stress scale (PSS) and 2) Bradford somatic inventory (BSI). Children: prematurity, perinatal death, low birth weight, macrosomia and neonatal distress.	15 days
Everard 2011 [52]	Australia	Total of women: 3,531 Stressed women: NR Non-stressed women NR Children: NR	Not reported	Participants were categorized in four groups: low; mild; moderate; and high stress score. Low stress score was used as the non-stressed group.	Maternal: Perceived Stress Scale (PSS) Children: preterm and birth weight.	20 weeks gestation
Chuang 2010 [50]	Taiwan	Stressed women: 46 Non-stressed women: 140 Children: NR	32.6	The authors categorized the working related stress to women during pregnancy into stressed women, who always perceived stress at work; and non-stressed women, who never or rarely perceived stress at work.	Maternal: a self-report questionnaire within 3 days after Delivery; the vitality (VT) and mental health (MH) subscales of the Taiwanese version of the short-form 36 (SF-36) were measured as self-reported psychological stress; finally, it was added ‘Do you feel stressed by working during pregnancy?’ to measure work stress. Children: the Comprehensive Developmental Inventory for Infants and Toddlers (CDIIT) diagnostic test conducted by physical therapists	2 years
Li 2010a [53]	Denmark	Women: NR Exposed children: 29,094 Unexposed children: 986,816	27–31	Children born to women who lost a close relative during pregnancy or up to 1 year before pregnancy. These children were included in the exposed cohort and other children were in the unexposed cohort.	Maternal and children: data from the Danish Civil Registration System Children: Hospital diagnosis of ADHD (ICD-10 code F90); or redeemed ADHD medication	3 years
Li 2010b [54]	Denmark	Women: NR Exposed children: 459 Unexposed children: 65.212	27–31	Prenatal stress was defined by being born to mothers who were bereaved by death of a close family member from one year before pregnancy until birth of the child.	Maternal: National health care system records Children: Body mass index (BMI) was calculated as weight (kg) / height (m)^2^. As the height and weight measurements were collected as part of routine school health examinations.	25 years
Whitehouse 2010 [55]	Australia	Stressed women: 490 Non-stressed women: 819 Children: 1,309	28.37	Stress was considered in the presence of two or more events during early (18 weeks) and late (34 weeks) pregnancy related to: pregnancy problems, death of a close friend or relative, separation or divorce, marital problems, problems with children, job loss (involuntary), partner's job loss (involuntary), money problems, residential move. The unexposed group was considered in the absence of the above.	Maternal: life stress inventory at both 18 and 34 weeks pregnancy Children: Language ability at the 10 year follow-up was assessed with the Peabody Picture Vocabulary Test-Revised (PPVT-R)	10 years
Xiong 2008 [56]	United States	Stressed women: 93 Non-stressed women: 127 Children: 299	18–35	High hurricane exposure was defined as having three or more of the eight severe hurricane experiences, such as feeling that one’s life was in danger, walking through floodwaters, or having a loved one died. The low stressed group was considered having less than three hurricane exposure	Maternal: Post-traumatic Stress Checklist (PCL)—Civilian Version; Edinburgh Depression Scale (EDS). Children: low birth weight (birth weight <2,500 grams) and preterm birth (gestational age <37 weeks)	Until childbirth
Dole 2003 [51]	United States	Stressed women: 1,462 Non-stressed women: 481 Children: 1,962	16–30	The authors categorized life event as an external stressor into low stress, medium-low stress, medium-high stress and high stress. We considered as the stressed group: medium-low stress, medium-high stress and high stress, while low stress was considered the non-stressed group.	Maternal: Psychosocial questionnaire (24–30 weeks’ gestation); telephone interview administered around 29 weeks’ gestation; hospital records. Children: Preterm birth was defined as less than 37 weeks’completed gestation.	4 years

*No.: number of participants at final analysis.

Xiong 2008[56] study evaluated hurricane exposure during childbearing; Li 2010a [53] and Li 2010b [54] studies dealt with grief during pregnancy. Whitehouse 2010 [55] study measured stressful life events, such as loss of a close person, divorce, marital problems and job loss (Table 3). Chuang 2011 [50] study presented work-related stress. Everard 2011[52] and Dole 2003 [51] studies considered perceived stress between maternal scores and psychological stress, respectively. Tandu-Umba 2014 [57] evaluated how maternal stress influence on fetal outcomes, such as prematurity, low birth weight, fetal death and neonatal distress (Table 3).

Therefore, all outcomes were related to birth weight [52,54,56]; preterm birth [51,52]; cognitive and motor aspects [50]; and language ability [55]. Furthermore, follow up ranged from delivery [56] to 13 years of age in children [54] (Table 3). However, two studies did not report any follow up period [51,52] (Table 3).

### Risk assessment

**Fig 2:**
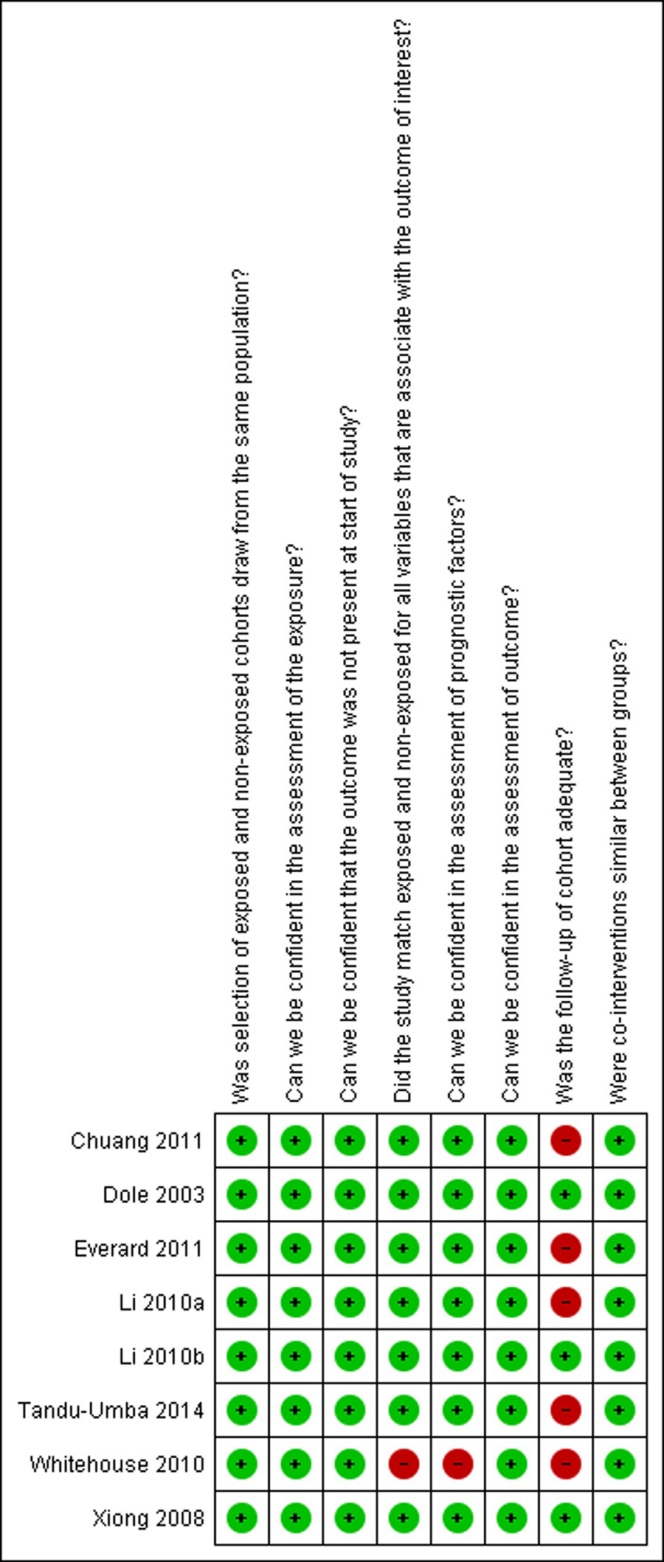
Risk of bias assessment.

Fig 2 described risk of bias assessment for cohort studies. The entire methodological quality of included studies was equally separated into unclear and low risk of bias categories. However, the major issue was the risk of bias related to follow-up in Chuang (2011), Everard, Li (2010a), Tandu-Umba (2014) and Whitehouse (2010), whose studies were unclear whether exposed and non-exposed group matched all variables associated with outcome measures and prognostic factor assessment.

### Outcomes

#### Low birth weight

**Fig 3:**
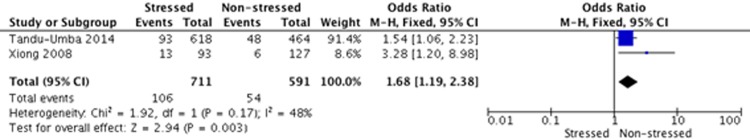
Meta-analysis of low birth weight.

Meta-analysis from two included studies (Tandu-Umba 2014; Xiong 2008) that involved 1,302 women showed a statistically significance difference favoring non-stressed group compared to antenatal stressed group (Odds ratio (OR) 1.68 [95% Confidential Interval (CI) 1.19, 2.38]). Therefore, there were more children with low birth weight born from stressed women compared to non-stressed women (Fig 3 and Table 4).

**Table 4:** Risk of bias assessment for cohort studies.

Author, year	Was selection of exposed and non-exposed cohorts drawn from the same population?	Can we be confident in the assessment of exposure?	Can we be confident that the outcome of interest was not present at start of study?	Did the study match exposed and unexposed for all variables that are associated with the outcome of interest or did the statistical analysis adjust for these prognostic variables?	Can we be confident in the assessment of the presence or absence of prognostic factors?	Can we be confident in the assessment of outcome?	Was the follow up of cohorts adequate?	Were co-Interventions similar between groups?
Tandu-Umba 2014	Definitely yes (low risk of bias)	Definitely yes (low risk of bias)	Definitely yes (low risk of bias)	Definitely yes (low risk of bias)	Definitely yes (low risk of bias)	Definitely yes (low risk of bias)	Probably no	Probably yes
Chuang 2011	Probably yes	Definitely yes (low risk of bias)	Definitely yes (low risk of bias)	Definitely yes (low risk of bias)	Definitely yes (low risk of bias)	Definitely yes (low risk of bias)	Probably no	Probably yes
Everard 2011	Definitely yes (low risk of bias)	Definitely yes (low risk of bias)	Definitely yes (low risk of bias)	Definitely yes (low risk of bias)	Definitely yes (low risk of bias)	Probably yes	Probably no	Probably yes
Whitehouse 2010	Definitely yes (low risk of bias)	Definitely yes (low risk of bias)	Definitely yes (low risk of bias)	Probably no	Probably no	Definitely yes (low risk of bias)	Definitely no (high risk of bias)	Probably yes
Li 2010a	Definitely yes (low risk of bias)	Definitely yes (low risk of bias)	Definitely yes (low risk of bias)	Definitely yes (low risk of bias)	Definitely yes (low risk of bias)	Definitely yes (low risk of bias)	Probably no	Probably yes
Li 2010b	Definitely yes (low risk of bias)	Definitely yes (low risk of bias)	Definitely yes (low risk of bias)	Definitely yes (low risk of bias)	Definitely yes (low risk of bias)	Definitely yes (low risk of bias)	Definitely yes (low risk of bias)	Probably yes
Xiong 2008	Definitely yes (low risk of bias)	Definitely yes (low risk of bias)	Definitely yes (low risk of bias)	Definitely yes (low risk of bias)	Definitely yes (low risk of bias)	Definitely yes (low risk of bias)	Definitely yes (low risk of bias)	Probably yes
Doler 2003	Definitely yes (low risk of bias)	Definitely yes (low risk of bias)	Definitely yes (low risk of bias)	Definitely yes (low risk of bias)	Definitely yes (low risk of bias)	Definitely yes (low risk of bias)	Definitely yes (low risk of bias)	Probably yes

All answers as: definitely yes (low risk of bias), probably yes, probably no, definitely no (high risk of bias).

#### Preterm labor

**Fig 4:**
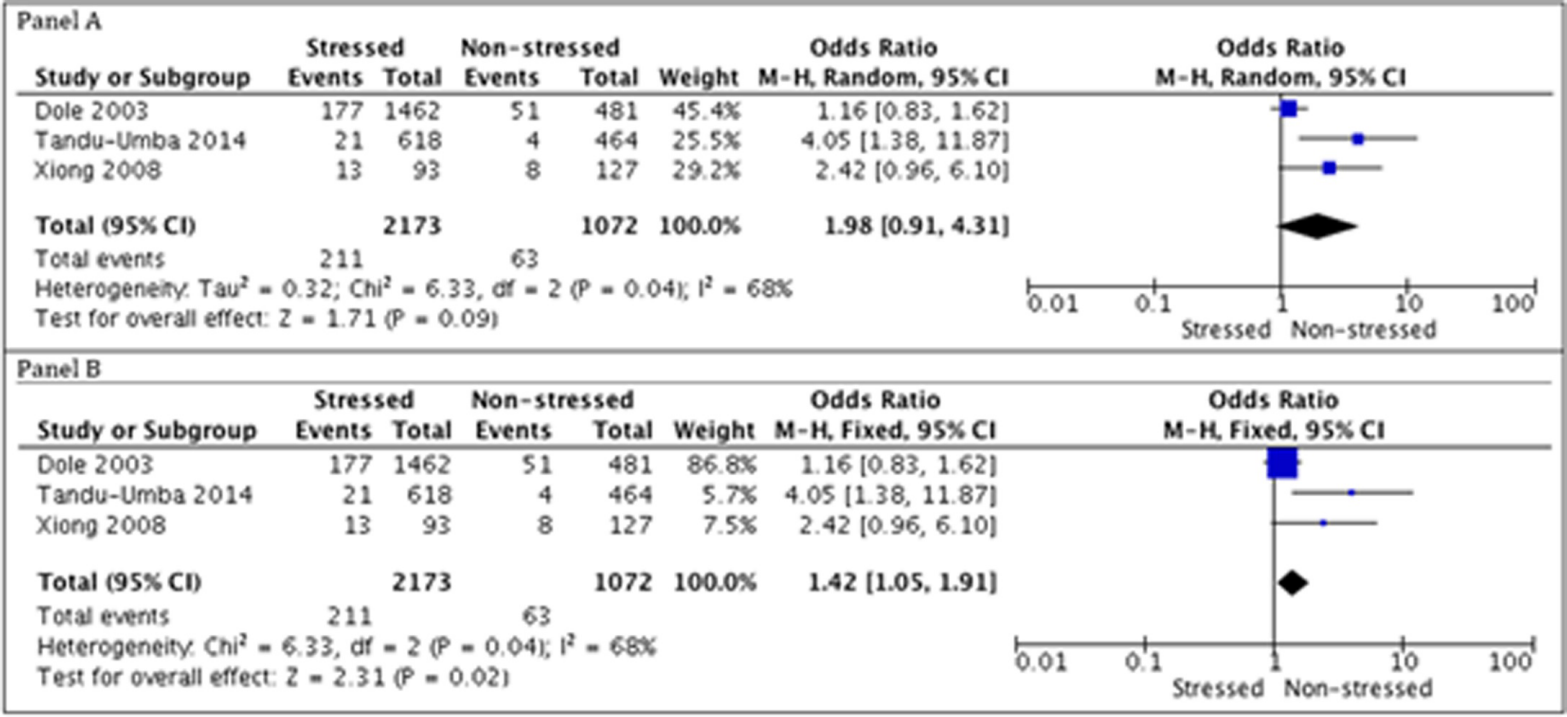
Meta-analysis of pre-term birth.

Meta-analysis from three included studies (Dole 2003; Tandu-Umba 2014; Xiong 2008) that had 3,245 women showed no statistically significance difference between non-exposed and exposed groups (OR 1.98 [95% CI 0.91 to 4.31]; I2 = 68%, p = 0.04). However, considering a fixed effect model in the sensitivity analysis, we found a statistically significance difference favoring non-stressed women group related to preterm birth rates (OR 1.42 [95% CI 1.05 to 1.91]; I2 = 68%, p = 0.04). Moreover, there was 1.42 more times preterm children born from stressed women compared to non-stressed one. According to a likely-case scenario, sensitivity analysis showed unsteadiness for such result; therefore, we considered that different effect models influenced results on preterm labor; consequently, showing an association between stress factors and higher preterm birth rates (Fig 4).

#### Language ability

There was a statistically significance difference favoring stressed group compared to antenatal non-stressed at early pregnancy, MD -1.24 [95% CI -3.22, 0.74]; late pregnancy MD -2.50 [95% CI -4.53, 0.47]; early and late pregnancy MD -2.38 [95% CI -4.44, -0.32] (Table 4 and S1 Appendix).

#### Fetal death

There was a statistically significance difference favoring non-stressed group compared to antenatal stressed one, OD 2.64 [95% CI 1.13, 6.18] (Table 4 and S2 Appendix).

#### Prevalence of overweight and obesity

The study of Withehouse (2010) described a prevalence of overweight and obesity, found statistically significant difference favouring antenatal non-stressed compared to stressed group within four time periods: i.e. from 10 to 13 years old: 10 years, RR 1.63 [95% CI 1.14, 2.35]; 11 years, RR 1.80 [95% CI 1.22, 2.65]; 12 years, RR 2.20 [95% CI 1.45, 3.34] and; 13 years RR 1.72 [95% CI 1.07, 2.76] (Table 4 and S3 Appendix).

## Discussion

### Main findings

The current study aimed to investigate whether there is a relationship between maternal stress exposure and fetal, neonatal or child development throughout a systematic review of cohort studies. Eight studies were included in the current review [50–57]. Studies showed clinical and methodological differences, establishing the veracity of the information.

### Strengths and limitation

Current evidence between maternal stress exposure and fetal, neonatal or child development were presented in the current review. There was a statistically significant difference favoring non-stressed group compared to antenatal stressed one, with regards to low birth weight; pre-term; overweight/obesity; and language ability. However, there were no statistically significant differences between groups when child’s motor development and attention deficit hyperactivity disorder (ADHD) were evaluated.

Such statements have been reinforced by these observational studies methodological quality due to well-formulated question; since minutiously literature search through electronic databases; selection, identification and data extraction was performed by two independent reviewers; besides critical appraisal of the included studies was made through measurement tool adapted by us [18].

### Relation to prior work

Antenatal stress has been associated with fetal weight [52,54,56] and preterm birth [51]. Li 2010b [54] study suggests severe pre-pregnancy stress is associated with an increased risk of overweight and obesity in later childhood. However, Xiong 2008 [56] and Everard 2011[52] studies observed that children from mothers, who have perceived maternal stress, presented an increased risk of having low birth weight. Antenatal stress was also related to changes in children development. For Chuang 2011[50] study, mothers, who perceived work-related stress, influenced child’s motor development later in life. Besides Li 2010a [53] study suggests severe stress during pregnancy may increase attention-deficit/hyperactivity disorder in offspring. However, Whitehouse (2010) [55] stated factors are perceived stress and stressful life events during pregnancy, such as vocabulary development within middle childhood.

### Implications

Based on data, we can infer that antenatal stress negatively influences fetal and children life. Although we also suggest further well-conducted studies with adequate sample size and longer follow-up time to confirm or refute our findings.

## Supporting information

S1 Appendix Representation of meta-analysis of language ability at 10 year. (DOCX)

S2 Appendix Representation of meta-analysis of perinatal death. (DOCX)

S3 Appendix Representation of meta-analysis of prevalence of overweight and obesity. (DOCX)

S1 Table PRISMA checklist. (DOC)
