# Supplementary figures and images for: Is the risk of low birth weight or preterm labor greater when maternal stress is experienced during pregnancy? A systematic review and meta-analysis of cohort studies
Source: PLoS One. 2018 Jul 26;13(7):e0200594. doi: 10.1371/journal.pone.0200594 (PMC6061976; doi:10.1371/journal.pone.0200594)

**S1 Appendix.** Representation of meta-analysis of language ability at 10 year


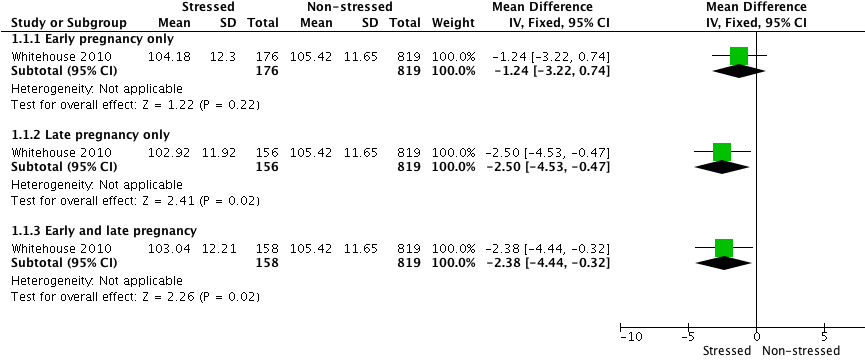

Supplement: S1 Appendix — (DOCX) [file pone.0200594.s001.docx]

**S2 Appendix.** Representation of meta-analysis of perinatal death


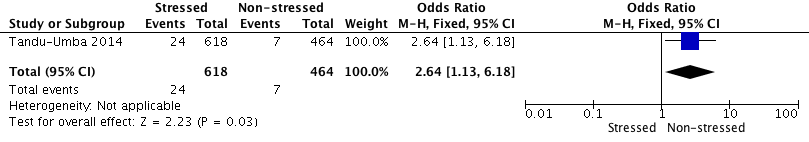

Supplement: S2 Appendix — (DOCX) [file pone.0200594.s002.docx]

**S3 Appendix.** Representation of meta-analysis of prevalence of overweight and obesity.


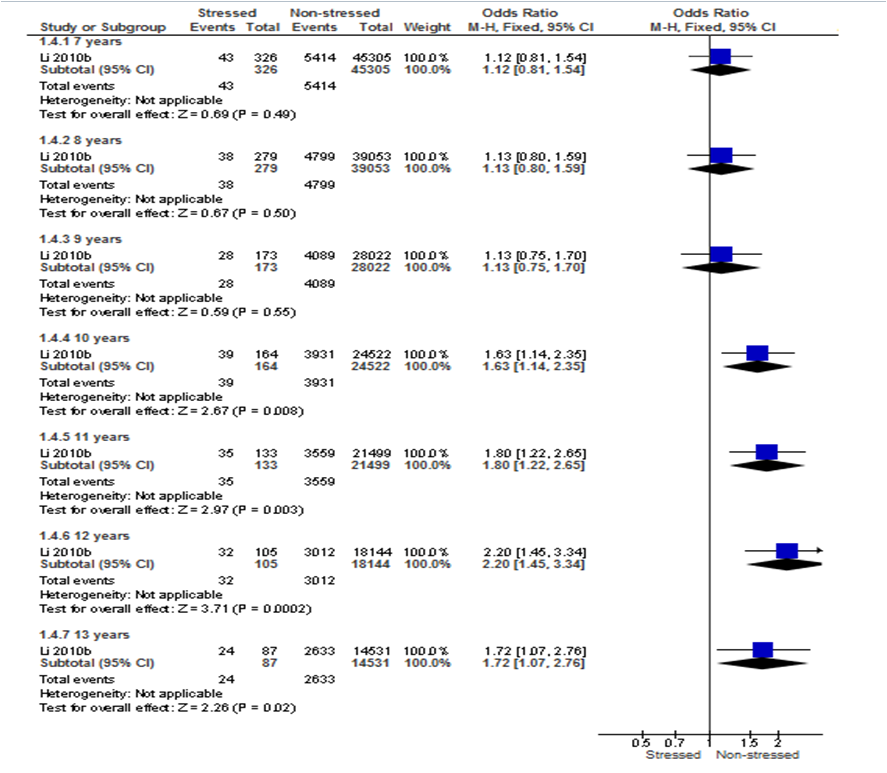

Supplement: S3 Appendix — (DOCX) [file pone.0200594.s003.docx]
